# The value of a preoperative physical therapy and home evaluation program in total joint arthroplasty

**DOI:** 10.1007/s00402-026-06244-0

**Published:** 2026-02-26

**Authors:** Kylie T. Callan, Maddison McLellan, Brandon Lung, Megan Donnelly, Leo Issagholian, William McMaster, Russell Stitzlein, Steven Yang

**Affiliations:** 1 University of California Irvine School of Medicine, Irvine, CA USA; 2 Department of Orthopaedic Surgery, University of California Irvine Health, Orange, CA USA; 3 Department of Orthopaedic Surgery, New York University Langone Health, New York, NY USA; 4 California University of Science and Medicine, Colton, CA USA

**Keywords:** Arthroplasty, Total hip arthroplasty, Total knee arthroplasty, Physical therapy

## Abstract

**Purpose:**

The implementation of prehabilitation prior to total joint arthroplasty (TJA) has been recognized to potentially decrease pain, reduce length of stay (LOS), and increase patient satisfaction. With longer wait times for surgery due to the COVID-19 pandemic contributing to deterioration of function, this study aims to assess the benefits of a physical therapy (PT) and home evaluation program to improve outcomes.

**Methods:**

A retrospective chart review of 130 primary THA and 124 primary TKA patients undergoing a new pre-operative PT and home safety evaluation program was performed to assess outcomes. Demographic data were compared to assess baseline characteristics. Pain was evaluated with inpatient/outpatient morphine milligram equivalents (MME) and VAS scores. Mobility was assessed using multiple measures by a physical therapist. Mean postoperative range of motion (ROM), overall complications, and non-home discharge was compared.

**Results:**

Of the 254 TJA patients, 67 (26%) patients underwent the prehabilitation program. Prehabilitation THA patients had statistically significantly higher Boston Activity Measure Post-Acute Care (AMPAC) scores on postoperative day 0, lower subjective VAS pain scores on the day of discharge, and greater 3-month postoperative ROM measurements. Prehabilitation TKA patients had statistically significantly less outpatient opioid MME pain requirements (*p* < 0.05). There were no significant differences in LOS, discharge destination, use of walking aids, or surgical complication rates.

**Conclusion:**

Prehabilitation programs prior to TJA may facilitate early postoperative mobility and improved pain relief through patient education and conditioning. In older patients with chronic pain, prehabilitation prior to TJA may contribute to lower pain scores and less opioid requirements.

## Introduction

Total joint arthroplasty (TJA) is a life-changing surgery that is frequently performed in the United States for quality of life, pain relief, and restoration of function to the affected joint [1, 2]. The annual number of TJAs is projected to grow along with the country’s aging population [3]. TJA is an intensive procedure and recent research has focused on enhancing recovery postoperatively and decreasing hospital length of stay (LOS) by a number of means including education, medication optimization, laboratory optimization, and hastening recovery [4]. Physical therapy (PT) is often utilized in TJA to promote recovery by strengthening previously compromised musculature and restoring function to the newly replaced joint [5]. Personalized PT care in the perioperative setting may aid in decreasing LOS, decreasing duration of prescription opioid use, and increasing early function [6, 7]. Typical focus is placed on rehabilitation and seldom is the regimen personalized despite a wide variability in the make and manufacturer of each prosthesis [8]. A thorough evaluation and needs assessment, including patients’ access to an accommodating home layout and help with recovery, is often performed perioperatively for TJA patients. This evaluation and subsequent tailoring of PT can give patients a greater quality of life following TJA [9].

PT is a common service in the inpatient setting and frequently utilized not just for TJA, though the literature has only increased regarding its use and efficacy in the last decade. Soeters et al. evaluated 126 TJA patients in an intervention study using personalized one-on-one PT instructions compared to group, generic instructions and found that patients with personalized instructions achieved discharge from PT a day earlier with fewer visits [6]. This suggests that close instruction and follow up by PT may help patients undergoing TJA with mobility and function, which can decrease LOS and improve perioperative outcomes. Rutenberg et al. found that earlier PT evaluation for patients following total knee arthroplasty (TKA) was associated with lower amounts of opioids prescribed during hospital stay, although there was no difference in gross range of motion (ROM) or muscle strength, which may suggest that PT is helpful for pain control [2]. Though several studies identified a net positive to employing PT, some studies display conflicting results. Yayac et al. reported increased costs with greater risk for short-term complications with the employment of personalized, home visit PT when compared to general outpatient therapy [10]. Short and medium-term outcomes of adverse events, ROM, and muscle strength are not grossly different depending on immediate vs. three-day postoperative PT evaluation [2]. Implementation of a sub-acute PT program also did not show any differences when compared to weekend-only programs in TJA patients [11]. In a randomized control trial consisting of 108 patients, Austin and colleagues reported similar functional improvements in patients undergoing structured, formal PT compared to informal, home therapy [1]. Despite insufficient evidence either in favor of or against a personalized, early PT regimen, there continues to be an increase in the use of PT criteria in the inpatient setting for discharge clearance and by the healthcare system overall [1, 12].

Healthcare optimization in order to save costs and avoid unnecessary procedures has led to a trend in investigating the optimal and most effective use of PT [4]. Decreased LOS and postoperative pain control are often the topics of discussion [7, 9, 13]. At this level one, academic institution, there has been a recent implementation of a novel preoperative PT and home safety evaluation program that allows for preoperative assessment of function, early education, and evaluation of the safety of postoperative discharge home that has not yet been assessed for efficacy. The aim of this study was to determine whether this program, especially the addition of the preoperative rather than postoperative home evaluation, affects postoperative outcomes for TJA patients. The authors of this study hypothesized that patients who received the intervention would see a more expeditious recovery in the form of early discharge, less use of opioid medications for pain, and higher postoperative functional scores.

## Methods

A retrospective chart review of primary TJA patients undergoing a newly implemented preoperative PT and home evaluation program between 2021 and 2022 at a single academic center was performed to assess postoperative outcomes. Patients were eligible for this preoperative PT and home evaluation program based on their insurance coverage. The prehabilitation group underwent a preoperative PT visit that included an assessment of their home features including presence of stairs, previous functional level, and presence of anyone in the home to assist the patient to determine safety of home discharge postoperatively, instruction on using mobility aids such as a walker, fall prevention education, and a home exercise program booklet with strengthening exercises based on which joint they were having replaced. The non-prehabilitation cohort was a group of control patients from the same time period who also underwent primary TJA but whose insurance did not cover preoperative PT and therefore received no preoperative counseling from a physical therapist. Patients received the same, standard care in the hospital regardless of group. Patients were discharged when they were deemed safe to do so by PT and the surgical team, with the same considerations regardless of prehabilitation status. Postoperatively, patients were seen by the surgeon at the same, standard intervals regardless of whether they received preoperative PT, at two, six, and 12 weeks postoperatively, corresponding to postoperative visits one, two, and three, at which point ROM, morphine milligram equivalents (MMEs), and use of mobility aids was recorded.

Demographic characteristics of both the prehabilitation and non-prehabilitation cohorts were collected from the chart, including age, gender, body mass index (BMI), American Society of Anesthesia (ASA) class, ethnicity, history of chronic pain, and history of opioid use. Baseline activity level was also assessed by reviewing the chart for preoperative ROM as measured visually during the preoperative visit physical exam, family support at home, ambulation status prior to surgery, ability to perform activities of daily living (ADLs) independently, and accessibility of home.

Data collected from the chart to assess outcomes included discharge destination, LOS, and complications including surgical site infection (SSI) excluding superficial SSI, deep vein thrombosis (DVT)/pulmonary embolism (PE), and postoperative neurologic, pulmonary, or cardiac complications.

Measurements of pain were assessed via MMEs given inpatient and at the first, second, and third postoperative visits and visual analog scores (VAS) recorded by nursing on postoperative day 0 (POD0) and discharge day. Mobility was assessed using PT metrics recorded in the chart including distance ambulated, gait assistance percentage, and Boston University Activity Measure for Post-Acute Care (AMPAC) scores on POD0 and discharge day. The amount of bathing and dressing assistance needed on discharge day were also collected from the occupational therapy notes.

Statistical analysis was performed using Chi-squared, Fisher’s exact test, t-test, and ANOVA. Bivariate logistic regression was used to evaluate influence of preoperative PT on outcome measures postoperatively. Statistics were performed with IBM SPSS Statistics, Version 26 (IBM Corp., Armonk, NY). Significance was defined as *p* < 0.05. De-identified data are available on reasonable request from the corresponding author, KTC.

## Results

### Total hip arthroplasty

#### Demographic data

There were 41 patients in the total hip arthroplasty (THA) prehabilitation cohort and 89 patients in the THA non-prehabilitation cohort. The mean age of patients in the prehabilitation group was significantly older (74.2 ± 6.3 vs. 64.5 ± 11.5, *p* < 0.001). There were more females in the prehabilitation cohort (25 (61.0%)) compared to the non-prehabilitation group (36 (40.4%)) (*p* = 0.038). BMI was similar across both groups (29.5 ± 6.3 vs. 29.4 ± 7.2, *p* = 0.906) as was ASA class distribution (Table 1). The prehabilitation cohort had significantly more patients with a history of chronic pain (22 (25.0%) vs. 40 (97.6%), *p* < 0.001). There was no significant difference in history of opioid use (16 (18.0%) vs. 11 (26.8%), *p* = 0.254).

**Table 1:** Demographics and medical history of THA no Pre-OP PT and Pre-Op PT patients

	No Pre-Op PT (*n* = 89)	Pre-Op PT (*n* = 41)	p
Age, mean ± SD	64.5 ± 11.5	74.2 ± 6.3	**< 0.001**
*Sex, n (%)*			**0.038**
Male	53 (59.6)	16 (39.0)	
Female	36 (40.4)	25 (61.0)	
*Medical history*			
BMI, mean ± SD	29.5 ± 6.3	29.4 ± 7.2	0.906
ASA Class, n (%)			0.153
I	4 (4.6)	0 (0)	
II	45 (51.7)	17 (41.5)	
III	38 (43.7)	24 (58.5)	
History of Chronic Pain, n (%)	22 (25.0)	40 (97.6)	**< 0.001**
History of Opioid Use Before Surgery, n (%)	16 (18.0)	11 (26.8)	0.254

significant values in bold

#### Baseline activity

At baseline, the prehabilitation cohort had significantly greater preoperative ROM (89.2 ± 17.3 vs. 96.6 ± 12.4, *p* = 0.019). There was no difference in family support at home (NP: 7 (100.0%) vs. PP: 34 (82.9), *p* = 0.573), prior ambulation independence (NP: 4 (57.1%) vs. PP: 28 (68.3%), *p* = 0.672), prior ability to perform ADLs independently (NP: 7 (100%) vs. PP: 41 (100.0%), *p* = 1), or house accessibility with wheelchair/walker (NP: 4 (57.1%) vs. PP: 22 (53.7%), *p* = 1) (Table 2).

**Table 2:** Baseline activity level of THA no Pre-op PT and Pre-Op PT patients

	No Pre-Op PT (*n* = 89)	Pre-Op PT (*n* = 41)	*p*
Preop ROM, mean ± SD	89.2 ± 17.3	96.6 ± 12.4	**0.019**
Spouse/family available to help at home, n (%)	7 (100.0)	34 (82.9)	0.573
Community ambulator without cane, n (%)	4 (57.1)	28 (68.3)	0.672
Performs ADLs independently, n (%)	7 (100.0)	41 (100.0)	–
House is walker/wheelchair accessible, n (%)	4 (57.1)	22 (53.7)	1.000

significant values in bold

#### Outcomes

There was no difference in rate of discharge home ((NP: 82 (92.1%) vs. PP: 37 (90.2%), *p* = 0.741) or LOS ((NP: 1.9 ± 1.1 vs. PP: 1.9 ± 1.2 *p* = 0.971). There was no difference in complications, including SSIs ((NP: 6 (6.7%) vs. PP: 1 (2.4%), *p* = 0.431), DVT/PE (NP: 1 (1.1%) vs. PP: 0 (0%), *p* = 1), or postoperative neurologic (none reported in either group), pulmonary (NP: 0 (0%) vs. PP: 3 (7.3%), *p* = 0.089), or cardiac complications (NP: 0 (0%) vs. PP: 4 (9.8%), *p* = 0.068).

There was no difference in MMEs between cohorts at any time point (Table 3). The distance walked was not significantly different on POD0 (NP: 82.9 ± 134.3 vs. PP: 62.8 ± 70.1, *p* = 0.289) or day of discharge (NP 280.6 ± 311.5 vs. PP 180.4 ± 132.1, *p* = 0.069). There was no difference in percentage of gait assistance on POD0 (NP: 28.5 ± 19.8 vs. PP: 31.7 ± 23.1, *p* = 0.433) or discharge day (NP: 8.2 ± 11.8 vs. PP: 11.0 ± 13.7, *p* = 0.247).

**Table 3:** Outcomes of THA no Pre-Op PT and Pre-Op PT patients

	No Pre-Op PT (*n* = 89)	Pre-Op PT (*n* = 41)	*p*
Discharge destination: home, *n* (%)	82 (92.1)	37 (90.2)	0.741
Length of stay days, mean ± SD	1.9 ± 1.1	1.9 ± 1.2	0.971
*Complications, n (%)*			
Surgical site infection	6 (6.7)	1 (2.4)	0.431
DVT/PE	1 (1.1)	0 (0)	1.000
Postop pulmonary complication	0 (0)	3 (7.3)	0.089
Postop cardiac complication	0 (0)	4 (9.8)	0.068
Postop neuro complication	0 (0)	0 (0)	–
*Morphine equivalents, mean ± SD*			
Inpatient	115.2 ± 110.7	86.6 ± 59.2	0.059
1 st postop	43.7 ± 179.9	68.2 ± 207.3	0.089
2nd postop	23.4 ± 146.9	60.0 ± 112.9	0.165
3rd postop	3.5 ± 32.4	44.2 ± 126.1	0.054
*Distance, mean ± SD*			
POD0	82.9 ± 134.3	62.8 ± 70.1	0.289
Discharge day	280.6 ± 311.5	180.4 ± 132.1	0.069
*Gait assistance %, mean ± SD*			
POD0	28.5 ± 19.8	31.7 ± 23.1	0.433
Discharge day	8.2 ± 11.8	11.0 ± 13.7	0.247
*Boston PAC score, mean ± SD*			
POD0	15.3 ± 3.4	16.8 ± 2.4	**0.006**
Discharge day	18.4 ± 2.2	18.8 ± 2.0	0.344
Discharge day bathing/dressing% assistance, mean ± SD	18.4 ± 14.2	14.6 ± 12.5	0.134
*VAS average, mean ± SD*			
POD0	4.0 ± 3.9	4.3 ± 3.0	0.693
Discharge day	4.2 ± 2.4	3.3 ± 2.3	**0.047**
*ROM, mean ± SD*			
Postop visit #1 (2 weeks)	97.5 ± 17.7	93.7 ± 14.1	0.716
Postop visit #2 (6 weeks)	93.1 ± 13.1	102.0 ± 15.0	0.125
Postop visit #3 (12 weeks)	96.5 ± 16.5	106.4 ± 14.6	**0.047**
Using walker/cane 1 st postop visit, n (%)	61 (79.2)	32 (80.0)	1.000

significant values in bold

In terms of Boston AMPAC score, on POD0, the prehabilitation cohort had significantly higher scores (NP: 15.3 ± 3.4 vs. PP: 16.8 ± 2.4, *p* = 0.006), but not at discharge (NP: 18.4 ± 2.2 vs. PP: 18.8 ± 2.0, *p* = 0.344). There was no difference in discharge day bathing/dressing assistance needed (NP: 18.4% ± 14.2 vs. PP: 14.6 ± 12.5, *p* = 0.134). In terms of VAS, there was no difference at POD0 (NP: 4.0 ± 3.9 vs. PP: 4.3 ± 3.0, *p* = 0.693) but the non-prehabilitation group had significantly higher scores at discharge (NP: 4.2 ± 2.4 vs. PP: 3.3 ± 2.3, *p* = 0.047). Postoperative ROM was significantly better in the prehabilitation group at postoperative visit three versus the non-prehabilitation group (NP 96.5 ± 16.5 vs. PP: 106.4 ± 14.6, *p* = 0.077). There was no difference at postoperative visit one or two (Table 3). There was no difference in terms of patients using a walker/cane at the first postoperative visit between cohorts (NP: 61 (79.2%) vs. PP: 32 (80.0), *p* = 1).

In bivariate logistic regression analysis, there was no association of preoperative PT and discharge destination (OR: 0.790, CI (0.218, 2.864), *p* = 0.719), SSI (OR: 0.346, CI: (0.040, 2.970), p: 0.333) DVT/PE (OR: 0, CI (0.000,0.000), *p* = 0.998), pulmonary complications, (OR: 0.000, CI (0.00, 0.000), *p* = 0.997), cardiac complications (OR: 0.000, CI: (0.000, 0.000), *p* = 0.996) or using a cane or walker at the first postoperative visit (OR 1.049, CI (0.406, 2.714), *p* = 0.921) (Table 4).

**Table 4:** Bivariate logistic regression for outcome variables for THA

	OR	95% CI	*p*
Discharge destination: home	0.790	(0.218, 2.864)	0.719
Surgical site infection	0.346	(0.040, 2.970)	0.333
DVT/PE	0.000	(0.000, 0.000)	0.998
Postop pulmonary complication	0.000	(0.000, 0.000)	0.997
Postop cardiac complication	0.000	(0.000, 0.000)	0.996
Using walker/cane 1 st postop visit	1.049	(0.406, 2.714)	0.921

In bivariate logistic regression analysis, preoperative PT was associated with higher Boston AMPAC scores on POD0 (USC B: 1.499, CI: (0.325, 2.674), *p* = 0.013). It was not associated with LOS, MMEs at any time point, distance ambulated, gait assistance, discharge day bathing/dressing assistance, VAS on POD0/discharge day or ROM postoperatively (Table 5).

**Table 5:** Bivariate linear regression for outcome variables in THA

	USC B	95% CI	*p*
Length of stay days	−0.008	(−0.426, 0.411)	0.971
Morphine equivalents inpatient	−28.516	(−64.983, 7.952)	0.124
Morphine equivalents 1 st postop	42.104	(25.032, 102.512)	0.189
Morphine equivalents 2nd postop	36.628	(−15.302, 88.558)	0.165
Morphine equivalents 3rd postop	40.742	(12.053, 69.432)	0.145
Distance POD0	−20.154	(−64.714, 24.407)	0.372
Distance discharge day	−100.248	(−200.619, 0.123)	0.050
Gait assistance % POD0	3.207	(−4.867, 11.282)	0.433
Gait assistance % discharge day	2.737	(−1.925, 7.399)	0.247
Boston PAC score POD0	1.499	(0.325, 2.674)	**0.013**
Boston PAC score discharge day	0.387	(−0.420, 1.195)	0.344
Discharge day bathing/dressing % assistance	−3.803	(−8.992, 1.385)	0.149
VAS average POD0	0.278	(−1.114, 1.669)	0.693
Discharge day VAS average	−0.878	(−1.756, 0.000)	0.050
ROM postop visit #1 (2 weeks)	−3.775	(−24.627, 17.077)	0.716
ROM postop visit #2 (6 weeks)	8.875	(−2.560, 20.310)	0.125
ROM postop visit #3 (12 weeks)	9.864	(−1.111, 20.838)	0.077

significant values in bold

### Total knee arthroplasty

#### Demographic data

There were 25 patients in the TKA prehabilitation group and 99 patients in the TKA non-prehabilitation group. At baseline, the prehabilitation cohort was significantly older than the non-prehabilitation cohort. (74.5 ± 7.2 vs. 68.7 ± 7.6, *p* = 0.001). Both the prehabilitation and the non-prehabilitation cohort were majority female (Table 6). There was no difference in baseline BMI between groups (P: 30.7 ± 8.3, NP: 31.9 ± 6.0, *p* = 0.412). Almost all patients in the prehabilitation group had a history of chronic pain (24 (96.0%) vs. 30 (31.6%)) (*p* < 0.001). There was no difference in history of opioid use between groups (P: 6 (24.0), NP: 14 (14.7), *p* = 0.364). There was no difference in baseline ASA between cohorts (Table 6).

**Table 6:** Demographics and medical history of TKA no Pre-OP PT and Pre-Op PT patients

	No Pre-Op PT (*n* = 99)	Pre-Op PT (*n* = 25)	*p*
Age, mean ± SD	68.7 ± 7.6	74.5 ± 7.2	**0.001**
*Sex, n (%)*			0.489
Male	36 (36.4)	7 (28.0)	
Female	63 (63.6)	18 (72.0)	
*Medical history*			
BMI, mean ± SD	31.9 ± 6.0	30.7 ± 8.3	0.412
ASA class, n (%)			0.483
I	0 (1.0)	0 (0)	
II	40 (41.2)	8 (32.0)	
III	54 (55.7)	17 (68.0)	
IV	2 (2.1)	0 (0)	
History of chronic pain, n (%)	30 (31.6)	24 (96.0)	**< 0.001**
History of opioid use before surgery, n (%)	14 (14.7)	6 (24.0)	0.364
Preop ROM, mean ± SD	105.9 ± 16.5	98.8 ± 13.9	0.051

significant values in bold

#### Outcomes

There was no difference in rate of discharge home ((NP: 89 (91.8%) vs. PP: 21 (84.0%)), *p* = 0.265) or LOS ((NP: 2.5 ± 1.9 vs. PP: 2.2 ± 1.4, *p* = 0.428). There was no difference in SSIs ((NP: 3 (3.1%) vs. PP: 1 (4.0%), *p* = 1.000), DVT/PE (none reported in either group), or postoperative neurologic (none reported in either group), pulmonary (NP: 1 (1.1%) vs. PP: 3 (12.0%), *p* = 0.104), or cardiac complications (NP: 2 (2.1%) vs. PP: 4 (16.0%), *p* = 0.089) (Table 7).

**Table 7:** Outcomes of TKA no Pre-Op PT and Pre-Op PT patients

	No Pre-Op PT (*n* = 99)	Pre-Op PT (*n* = 25)	*p*
Discharge destination: home, *n* (%)	89 (91.8)	21 (84.0)	0.265
Length of stay days, mean ± SD	2.5 ± 1.9	2.2 ± 1.4	0.428
*Complications, n (%)*			
Surgical site infection	3 (3.1)	1 (4.0)	1.000
DVT/PE	0 (0)	0 (0)	–
Postop pulmonary complication	1 (1.1)	3 (12.0)	0.104
Postop cardiac complication	2 (2.1)	4 (16.0)	0.089
Postop neuro complication	0 (0)	0 (0)	–
*Morphine equivalents, mean ± SD*			
Inpatient	76.5 ± 109.0	70.5 ± 32.9	0.648
1 st postop	546.6 ± 379.7	220.6 ± 139.0	**< 0.001**
2nd postop	246.2 ± 460.5	66.0 ± 119.2	**0.001**
3rd postop	112.1 ± 325.0	62.5 ± 152.7	0.471
*Distance, mean ± SD*			
POD0	40.9 ± 60.4	53.3 ± 68.0	0.378
Discharge day	96.2 ± 106.4	203.3 ± 126.0	**0.023**
*Gait assistance %, mean ± SD*			
POD0	27.0 ± 13.0	30.0 ± 20.4	0.389
Discharge day	16.0 ± 14.2	9.0 ± 13.1	**0.020**
*Boston PAC score, mean ± SD*			
POD0	16.1 ± 2.9	16.8 ± 2.0	0.265
Discharge day	18.4 ± 2.6	18.5 ± 1.6	0.811
Discharge day bathing/dressing % assistance, mean ± SD	15.1 ± 15.5	17.0 ± 11.9	0.576
*VAS average, mean ± SD*			
POD0	4.0 ± 2.9	4.8 ± 3.1	0.224
Discharge day	4.1 ± 2.3	3.7 ± 2.5	0.435
*ROM, mean ± SD*			
Postop visit #1 (2 weeks)	91.9 ± 13.4	92.8 ± 9.2	0.726
Postop visit #2 (6 weeks)	102.0 ± 12.9	105.0 ± 11.3	0.292
Postop visit #3 (12 weeks)	105.4 ± 10.8	129.1 ± 10.7	**0.045**
Using walker/cane 1 st postop visit, n (%)	73 (83.0)	23 (92.0)	0.354

significant values in bold

The prehabilitation cohort had significantly lower MMEs at postoperative visit one and two (Table 7) but there was no difference while inpatient or at postoperative visit three (Table 7). There was no difference in distance ambulated on POD0 (NP: 40.9 ± 60.4 vs. PP: 53.3 ± 68.0, *p* = 0.378), but the prehabilitation cohort ambulated significantly longer distances on discharge day (NP 96.2 ± 106.4 vs. PP 203.3 ± 126.0, *p* = 0.023). There was no difference in gait assistance at POD0 (NP: 27.0 ± 13.0 vs. PP: 30.0 ± 20.4, *p* = 0.389), but the prehabilitation cohort required more gait assistance on discharge day (NP: 9.0 ± 13.1 vs. PP: 16.0 ± 14.2, *p* = 0.020). There was no difference in bathing/dressing assistance (Table 8).

**Table 8:** Bivariate logistic regression for outcome variables in TKA

	OR	95% CI	*p*
Discharge destination: home	0.472	(0.130, 1.716)	0.254
Surgical site infection	1.292	(0.129, 12.977)	0.828
Postop pulmonary complication	3.682	(1.258, 12.811)	0.401
Postop cardiac complication	8.762	(1.504, 24.046)	0.634
Using walker/cane 1 st postop visit	2.363	(0.503, 11.112)	0.276

There was no difference in Boston AMPAC scores or VAS average on POD0 or discharge day (Table 7). There was no difference in ROM at postoperative visits one or two (Table 7) or use of a walker/cane at the first postoperative visit (NP: 73 (83.0), P: 23 (92.0), *p* = 0.354). However, the prehabilitation cohort had significantly greater ROM at postoperative visit three (NP: 105 (10.8), P: 129.1 (10.7), *p* = 0.045).

In bivariate logistic regression analysis, there was no association of preoperative PT and discharge destination (OR: 0.472, CI (0.130, 1.716), *p* = 0.254), SSI (OR: 1.292, CI: (0.129, 12.977), p: 0.828), using a cane or walker at the first postoperative visit (OR: 2.363, CI (0.503, 11.112), *p* = 0.276) (Table 8) or postoperative pulmonary (OR: 8.762, CI (1.504, 24.046), *p* = 0.401) or cardiac complications (OR: 8.762, CI: (1.504, 24.046), *p* = 0.634).

In bivariate logistic regression analysis, preoperative PT was associated with lower MMEs needed at postoperative visit one (USC B: −326.030, CI: (−479.557,172.504), *p* < 0.001) as well as more gait assistance needed on discharge day (USC B: 7.053, CI: (1.115, 12.990), *p* = 0.02). It was also associated with greater distance ambulated on discharge day (USC B: 52.642, CI: (29.062, 304.778), *p* = 0.045) as well as greater ROM at postoperative visit three (USC B: 5.180, CI: (1.650, 20.290), *p* = 0.035). It was not associated with LOS, MMEs inpatient or at postoperative visits two or three, distance ambulated on POD0, Boston AMPAC scores, discharge day bathing/dressing assistance, VAS, or ROM at postoperative visits one or two (Table 9).

**Table 9:** Bivariate linear regression for outcome variables in TKA

	USC B	95% CI	*p*
Length of stay days	−0.325	(−1.133, 0.484)	0.428
Morphine equivalents inpatient	−5.971	(−49.793, 37.851)	0.788
Morphine equivalents 1 st postop	−326.030	(−479.557, −172.504)	**< 0.001**
Morphine equivalents 2nd postop	−180.206	(−364.883, 4.472)	0.056
Morphine equivalents 3rd postop	−49.588	(−185.486, 86.310)	0.471
Distance POD0	12.396	(−15.349, 40.141)	0.378
Distance discharge day	52.642	(29.062, 304.778)	**0.045**
Gait assistance % POD0	2.981	(−3.849, 9.811)	0.389
Gait assistance % discharge day	7.053	(1.115, 12.990)	**0.020**
Boston PAC score POD0	0.691	(−0.532, 1.915)	0.265
Boston PAC score discharge day	0.133	(−0.972, 1.239)	0.811
Discharge day bathing/dressing % assistance	1.884	(−4.764, 8.531)	0.576
VAS average POD0	0.799	(−0.495, 2.093)	0.224
Discharge day VAS average	−0.415	(−1.464, 0.634)	0.435
ROM postop visit #1 (2 weeks)	0.816	(−4.825, 6.458)	0.775
ROM postop visit #2 (6 weeks)	3.011	(−8.647, 2.624)	0.292
ROM postop visit #3 (12 weeks)	5.180	(1.650, 20.290)	**0.035**

significant values in bold

## Discussion

This study found that, in the THA group, PT patients were older, were more likely to be female, were more likely to have chronic pain, had higher preoperative ROM, had higher Boston AMPAC scores on POD0, had lower VAS pain scores on day of discharge, and had increased ROM at postoperative visit three. For the TKA group, PT patients were older, were more likely to have a history of chronic pain, had lower early outpatient MME requirements postoperatively, ambulated farther on discharge day, needed higher gait assistance percentage on day of discharge, and had greater ROM at postoperative visit three.

In this study, it was determined that both THA and TKA patients who had preoperative PT had improved ROM at postoperative visit three. This is somewhat consistent with what has been shown in the literature. A randomized control trial that had THA and TKA patients undergo twice-weekly therapy sessions preoperatively found that those who participated had significant improvements in knee ROM [14]. A study in Rome found that patients who participated in PT for a month before THA had increased hip external rotation preoperatively [15]. One single-blind randomized control trial that had patients complete an intensive telerehabilitation program prior to TKA found that there was a significant increase in knee flexion ROM for TKA patients six weeks postoperatively [16]. A meta-analysis by Chen and colleagues that examined several studies that employed preoperative exercise prior to TKA found a significant difference in knee ROM but no significant differences in isolated knee flexion or knee extension [17]. However, another clinical trial by Beaupre and colleagues examining a four-week exercise program prior to TKA found no difference in ROM or strength after the program or at any point postoperatively, up to six months [18]. Additionally, in another study, no preoperative or up to three-month postoperative differences in ROM were seen comparing patients who underwent a six-week preoperative rehabilitation program prior to primary TKA [19]. While the effect might not be as consistent across all types of PT programs, it is likely that increased PT preoperatively sets patients up for success postoperatively and allows them to regain more ROM. Optimizing ROM is key to getting patients back to previous levels of function, and therefore improving ROM by pursuing PT should be prioritized.

In terms of pain, the prehabilitation THA patients in this study had lower pain scores on day of discharge and the prehabilitation TKA patients had lower MME requirements outpatient postoperatively. The effects of preoperative programs on pain in the literature are inconclusive. A study on THA patients in Rome found that patients who underwent a month of preoperative PT had decreased VAS scores preoperatively and at one and three months postoperatively [15]. Another study examining TJA patients found that prehabilitation patients had decreased pain six weeks postoperatively, but no difference at three months [20]. However, Beaupre’s study of primary TKA patients undergoing a preoperative exercise regimen found no difference in pain after their four-week program or at any point postoperatively [18]. In Chen’s meta-analysis examining outcomes of TKAs after prehabilitation programs, there was no difference in pain scores between prehabilitation and non-prehabilitation groups [17]. On the other hand, a meta-analysis evaluating 35 studies that examined the effects of preoperative exercise programs found that THA patients who underwent preoperative programs had decreased pain relative to controls, but this effect was still not seen in TKA patients [21]. Overall, the differences in pain postoperatively in some variables but not all were somewhat consistent with the hypothesis that patients with prehabilitation would see a greater decrease in pain and less opioid use postoperatively. It is unsurprising that patients may have the same pain medication requirements inpatient, as they had the same invasive procedure performed. It is encouraging that there was any difference in VAS scores inpatient, given the recency of surgery and the relativity of the scores. After the initial healing, for outpatient MMEs, this study was limited to relying on the amount that was prescribed for symptoms, as there is not the same log of what the patient actually took, which may limit the ability to further elucidate any differences in the smaller sample size.

In regards to functional scores and outcomes postoperatively, in this study the THA prehabilitation group had higher Boston AMPAC scores on day of discharge, and TKA patients ambulated further on day of discharge but had a higher gait assistance percentage on day of discharge as compared to controls. These results were somewhat mixed compared to the hypotheses that prehabilitation patients would have improved functional outcomes as illustrated by increased gait distance, increased Boston AMPAC scores, and decreased gait assistance. However, the literature is also variable in terms of these outcomes. A randomized control trial that saw that TJA patients that underwent prehabilitation had increased knee ROM found that this had no impact on functional outcomes or quality of life scores [14]. Beaupre’s study found no difference in functional scores after their four-week program or at any point postoperatively [18]. Chen’s meta-analysis saw no difference in WOMAC stiffness scores following TKA for patients who had undergone prehabilitation regimens [17]. Additionally, there was no significant difference in KOOS scores up to 3 months postoperatively in primary TKA patients who had undergone six weeks of prehabilitation [19]. Soeters and colleagues evaluated WOMAC scores six weeks postoperatively after THA or TKA and found no difference between those who had a one-on-one PT evaluation preoperatively and those who did not [6]. A longer-term follow up of patients after THA found that although there were minor differences in favor of PT at three months postoperatively, there were no significant differences in functional scores or other functional measures at 12 months postoperatively [22]. Another preoperative program for frail patients prior to THA found that preoperatively it improved chair-rise time and timed up-and-go, but there were no significant differences postoperatively, including in time to reach functional independence [23]. Overall, while the results from this study are encouraging, there has been no consistent data that points to prehabilitation leading to definitively increased functionality postoperatively, which perhaps indicates that more personalized PT regimens are required.

This study found that there were no associations between preoperative PT and decreased LOS or discharge destination. This lack of measurable impact on LOS is in line with what has been seen by some other studies. Beaupre’s study found that patients who underwent prehabilitation used decreased resources postop and had shorter LOS, but these differences were not significant [18]. Soeters and colleagues evaluated the effect of a one-on-one, one-time PT meeting preoperatively prior to THA or TKA and found that while prehabilitation patients were ready to discharge sooner from a PT perspective, there was ultimately no difference in hospital LOS [6]. Two studies examining the effect of preoperative PT on frail THA patients saw no difference in hospital LOS [23, 24]. On the other hand, Huang and colleagues performed a randomized control trial to determine the effect of a home exercise program and found that their four-week, at-home program led to decreased LOS for TKA patients [25]. Overall, there are many variables that ultimately impact hospital LOS, and especially for operations such as total joints that require relatively short LOS postoperatively, there are likely other factors playing a larger role than PT readiness.

### Limitations

This study has several limitations and the results should be interpreted in the context of these. First, this is a single-center, retrospective study. All of the patients were treated at one surgical center in one geographic location. This was a relatively small sample size of patients. Additionally, as this was a retrospective study, patients were not randomized to the treatment group. Most often, whether patients participated in preoperative PT was dictated by which insurance they had and whether it would be covered financially. Differences were also present in demographics, as the PT group was significantly older than the non-PT group. These factors do not allow for a controlled comparison between the two. Finally, outcome measures are limited by what information is in the chart, such as PT notes, and many variables are measured by proxy (e.g. ambulation distance as a marker of function) as thorough surveys for comparison would need to be considered and implemented in a prospective fashion.

## Conclusion

In terms of the aim of this study, to determine whether a preoperative PT evaluation and regimen affects postoperative outcomes for TJA patients, and the hypothesis, that patients who received early PT evaluation would see a more expeditious recovery in the form of early discharge, less use of opioid medications for pain, and higher postoperative functional scores, the results were mostly in agreement but somewhat mixed. There was no difference in discharge destination or hospital LOS. The THA patients with preoperative PT had lower VAS pain scores on day of discharge, and the TKA patients had lower early outpatient MME requirements. Both the THA and TKA patients with preoperative PT had greater ROM at postoperative visit three. The THA patients had higher Boston AMPAC scores on POD0 and the TKA patients ambulated farther on discharge day, but needed higher gait assistance. Overall, preoperative PT has the potential to increase patient satisfaction and function by increasing ambulation distance, ROM, and functional scores and decreasing postoperative pain, but it must be specifically designed and implemented in order to optimize the benefits for all patients.

## Data Availability

Data are available upon reasonable request from the corresponding author, KTC.
